# Effect of Salt Reduction on Consumer Acceptance and Sensory Quality of Food

**DOI:** 10.3390/foods6120103

**Published:** 2017-11-27

**Authors:** Ulla Hoppu, Anu Hopia, Terhi Pohjanheimo, Minna Rotola-Pukkila, Sari Mäkinen, Anne Pihlanto, Mari Sandell

**Affiliations:** 1 Functional Foods Forum, University of Turku, 20014 Turku, Finland; ulla.hoppu@utu.fi (U.H.); anu.hopia@utu.fi (A.H.); terhi.pohjanheimo@utu.fi (T.P.); minna.rotola-pukkila@utu.fi (M.R.-P.); 2 Natural Resources Institute Finland (LUKE), 31600 Jokioinen, Finland; sari.makinen@luke.fi (S.M.); anne.pihlanto@luke.fi (A.P.)

**Keywords:** salt, reduction, food, taste, consumer, sensory

## Abstract

Reducing salt (NaCl) intake is an important public health target. The food industry and catering services are searching for means to reduce the salt content in their products. This review focuses on options for salt reduction in foods and the sensory evaluation of salt-reduced foods. Simple salt reduction, mineral salts and flavor enhancers/modifiers (e.g., umami compounds) are common options for salt reduction. In addition, the modification of food texture and odor-taste interactions may contribute to enhanced salty taste perception. Maintaining consumer acceptance of the products is a challenge, and recent examples of the consumer perception of salt-reduced foods are presented.

## 1. Introduction

High dietary salt (NaCl) intake is a significant risk factor for hypertension and thus a common public health challenge worldwide [1]. The World Health Organization recommends that adults should consume less than 2 g of sodium (5 g of salt) per day [2]. The current intake exceeds the recommended levels—for example, in selected European countries, the average salt intake of women is 7.3–10 g/day and that of men is 9.4–13.3 g/day [3]. The World Health Organization (WHO) global action plan for the prevention of non-communicable diseases aims to attain a 30% reduction in the mean population intake of salt [4]. Many countries have initiated programs targeting salt reduction [5].

In many European countries, home cooking has been declining, and only a very small part of sodium intake comes from household salt intake [6]. Processed foods in general (e.g., bread and bakery products, processed meat products, and cheese) contribute markedly to sodium intake [7]. Ready meals may have high salt levels [8], and currently, people eat more frequently outside the home (e.g., restaurants, fast-food outlets, and workplace canteens), where the salt content of meals may also be high [9]. Thus, for an ordinary consumer, reducing salt intake is a challenge because the choice of low-salt options in the market may be limited. Therefore, the role of the food industry and food services is critical, but they may have fears regarding reduced consumer preference and thus sales of low-salt products. In addition to taste, salt also contributes to other sensory characteristics, such as the aroma profile of foods, by increasing the volatility of aroma compounds, or mouthfeel, by affecting the lubricating properties of saliva [10]. Having an effect, for example, on the water holding capacity of proteins and water activity, salt has an effect on technological characteristics such as texture and microbiological safety of foods [11,12].

This review will focus on the perception of salty taste and the effects of salt reduction or modification of taste for the sensory perception of salty foods. Maintaining consumer preference is critical in developing low-salt products, and thus, the focus will be on the recent publications (mainly since 2010) on consumer acceptance and liking of salt-reduced/modified food products among adults.

## 2. Salt Taste Perception and Taste Interactions

### 2.1. Taste Receptors

Humans perceive five different taste modalities (bitter, sweet, umami, sour and salty). Taste receptors for bitter (TAS2R), as well as umami and sweet, are G-protein-coupled receptors [13]. During recent years, in particular, the perception of bitter taste has been studied actively, and the genetic variation in bitter taste receptors (e.g., TAS2R38) has been shown to be associated with individual differences in the sensitivity to specific bitter compounds and preference and consumption for certain foods, such as bitter-tasting vegetables [14,15]. 

Salt taste receptors have been studied much less, and the proposed receptor for salt taste is the epithelial sodium channel ENaC [16]. Genetic variation in the TRPV1 and SCNN1B genes has been suggested to modify salt taste perception in humans [17]. Heritability studies have suggested that genetics plays a smaller role in determining individual differences in the recognition thresholds for saltiness compared with those for sourness [18]. Recent findings have also suggested that multiple mechanisms could underlie the Amiloride-insensitive salt responses in type III taste cells [19], thus providing new insights into understanding the mechanisms underlying salt taste perception.

### 2.2. Salt Taste Sensitivity and Preference

Salt taste sensitivity and correlation to liking and intake also remains contradictory. In the USA, Hayes et al. [20] reported that females and males differed in their salt perception, and the impact of saltiness on liking varied across the food type. In Korea, men were found to have significantly higher thresholds and preferences for salty taste than women [21]. The salt detection threshold differed significantly between males and females, but individual salt detection and recognition thresholds had no significant effect on consumer acceptability and purchase intent scores of reduced-sodium vegetable soups [22]. Lucas et al. [23] found that the detection and recognition thresholds of NaCl were not associated with perceived saltiness, liking or intake of the food sample. Furthermore, the liking of salty foods differed in the sensory laboratory or dining room environment [23]. Therefore, it is not possible to predict the liking or consumption of salty foods based on a simple salt sensitivity test. 

Whether the liking of specific culture-specific salty foods may be an indicator of salt intake remains also open. Among Japanese subjects, the self-reported preference for salt taste in miso soup was associated with daily sodium intake [24]. It is also debatable regarding how easy it is to adopt to a low-sodium diet in general, and the studies have conflicting findings that could be explained by different study populations, the duration of the diet, and the test samples, among other factors. In a controlled low-sodium diet, no effect of reduced salt exposure over weeks on salt taste responses was observed [25]. Furthermore, sodium supplementation as capsules for four weeks did not change the salt taste responses, suggesting that preference for saltiness is independent of bodily sodium levels [25]. The simple salt taste preference test (NaCl in different concentrations in water solution) may have different results from actual salt level liking of complex foods and dishes as reviewed in later chapters. 

### 2.3. Flavor Interactions

The studies focusing on single salt taste quality as evaluated in taste thresholds and preferences of NaCl-water solutions in sensory laboratory may be misleading because, in foods, many different taste qualities are perceived simultaneously. When different taste qualities are mixed, several interactions (both enhancement and suppression) may occur, also depending on the food matrix [11]. Binary interactions have been studied in mixtures of aqueous solutions, and it has been shown that sour acids enhanced saltiness, and salts and sweeteners suppressed bitterness, as reviewed by Wilkie and Capaldi Phillips [26]. Therefore, if salt is reduced in food, other taste qualities, such as bitterness, may become more prevalent. Novel sensory evaluation techniques, such as the temporal dominance of sensations may provide new insights into multiple combinations of tastes [27].

In addition to the five primary taste qualities, the sensitivity to specific fatty acids and perception of fat may differ individually [28]. Fat and salt are a common combination in foods. The lipid content of a sample (oil-in-water emulsions) can affect saltiness perception [29]. Recently, Bolhuis et al. [30] reported that in tomato soups with different fat and salt contents, salt and fat affected pleasantness separately, with salt having the strongest effect.

## 3. Salt Reduction in Food

### 3.1. Overview of Previous Reviews

As salt reduction has been a topical issue, many thorough reviews have been published in recent years. Sodium reduction from a food industry perspective, in general, has been covered by some reviews [31,32]. Doyle and Glass [33], in turn, noted the safety issues (preservation and microbiological safety) in sodium reduction. Belz et al. [34], as well as Silow et al. [35], focused on salt reduction in bread and bakery products and dealt with both technological and qualitative challenges. Salt in bread from a European perspective was reviewed by Quilez and Salas-Salvado [36]. Sodium reduction in meat products has also been covered [37,38]. The review by Inguglia et al. [38] also listed some examples of commercially available products for sodium reduction for the food sector. 

**Figure 1:**
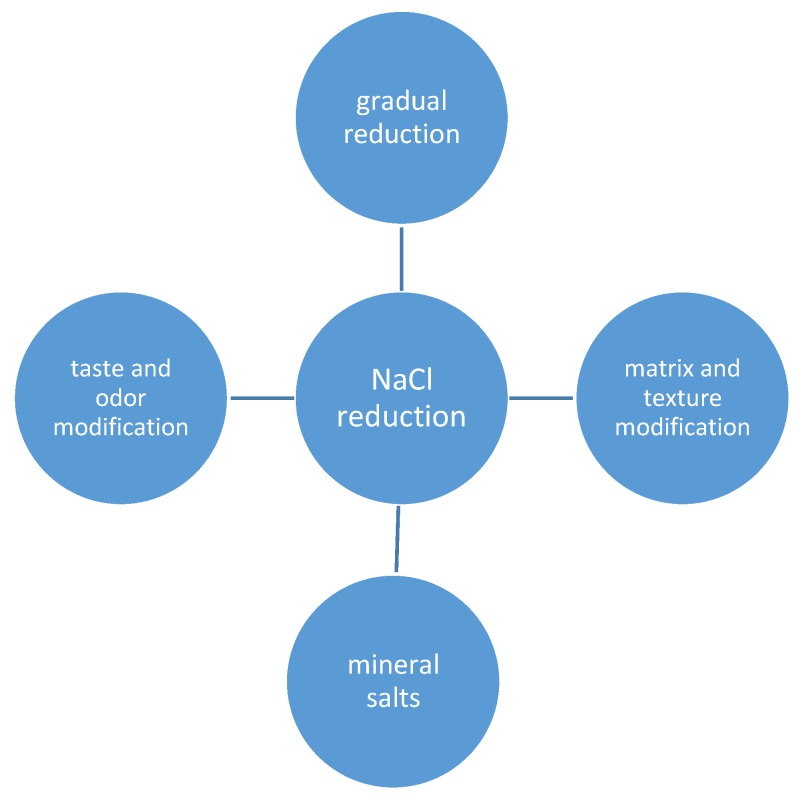
Overview of options for salt (NaCl) reduction in foods covered in this review.

Jaenke et al. [39] conducted a systematic review and meta-analysis of salt-reduced foods, containing tables of previous studies on salt reduction or replacement (mainly KCl or soy sauce). According to Jaenke et al. [39], consumer acceptance may be maintained even after very significant reductions of the salt content, but the results may differ between product categories (e.g., bread, cheese, and meat products). From the perspective of flavor, Liem et al. 2011 [11] discussed the sensory role of sodium, taste interactions and reduction of sodium, considering palatability. Since 2011, many new articles have been published, some of which are outlined in the present review (Figure 1). focusing on the sensory evaluation and consumer preferences of salt-reduced foods.

### 3.2. Simple Salt Reduction

Few examples of recent studies concerning different options for salt reduction are reviewed here. The most obvious and simplest option is to reduce the salt content of the products. The levels of salt reduction between products, as well as study protocols in sensory laboratories or real-life conditions, have varied, and some examples are discussed here. A consumer panel in a sensory laboratory evaluated reduced-sodium cheddar and mozzarella cheeses, and it was found that consumers could distinguish a 30% salt reduction [40]. In meat products evaluated by a trained sensory panel, the sensory parameters most affected by the salt reduction were salty taste, juiciness and texture. In sausages and ham, moderate salt reduction did not have a significant effect on the sensory quality, while bacon and salami were significantly affected after a moderate reduction [41].

Consumer tests measured the ability to detect differences in reduced-sodium bread and control bread (two-alternative forced-choice testing), acceptability including overall liking (nine-point hedonic scale) and purchase intent, and it was found that reducing sodium levels by up to 30% in bread did not affect consumer liking or purchase intent of the product [42]. When subjects consumed a buffet-style breakfast on weekdays for four weeks and received either regular bread or bread with gradually lowered salt content, 50% salt reduction in bread did not decrease bread consumption or affected the choice of sandwich fillings [43]. On the other hand, Antunez et al. [44] reported a large heterogeneity in consumer hedonic reactions to salt reduction in bread, and they noted the importance of consumer segmentation to understand consumer reactions to salt reduction.

In a 16-week longitudinal study, no overall difference in liking for low-sodium tomato juice at the final taste test was observed, but gradual salt reduction was more acceptable than the abrupt salt reduction [45]. Individual differences in hedonic sensitivity for salt and in the motivation to reduce salt intake may be challenging to reduce salt intake, and strategies targeted to specific consumer groups may be needed [45]. Multiple exposures have generally been shown to increase the liking of novel flavors and foods. The liking of soups varying in salt content has also been investigated, and it was shown that simple repeated exposure to the taste of the no-added salt soup was sufficient to increase liking [46]. 

### 3.3. Mineral Salts

A second option is to use mineral salts where sodium has been replaced by potassium, magnesium or calcium. However, the flavor characteristics of these salts may often be evaluated as negative by consumers. KCl may be associated with unpleasant side tastes such as bitter, metallic and chemical [47]. Individual variation in the perception of non-salty tastes for KCl was also observed to be large [47]. 

Therefore, regarding sensory properties, usually only a portion of NaCl may be replaced by other salts. For example, in reduced-fat mortadella, the sensory acceptance was best when 50% NaCl was replaced by 25% KCl and 25% CaCl_2_; however, calcium chloride reduced the emulsion stability and cooking yield [48]. In dry-cured ham, all sensory attributes were affected, yielding poorer scores in the case of hams containing CaCl_2_ and MgCl_2_. Hams salted with KCl + NaCl were rated equally to control hams, except for worse taste, probably due to the potassium contribution to a bitter taste [49].

In reduced-sodium cheddar-style cheese, both CaCl_2_ and MgCl_2_ produced considerable off-flavors (bitter, metallic, soapy), while cheeses containing KCl + NaCl did not differ significantly from control, as evaluated by descriptive sensory analyses of a trained panel [50]. Some other flavoring extracts and compounds have been used in combination with KCl to improve the sensory attributes—for example, yeast extract [51]. 

### 3.4. Umami Taste Compounds

Umami taste is described as brothy or beefy, and the taste sensation comes from free amino acids glutamic acid (glutamate) and, to a lesser extent, aspartic acid [52]. Umami taste can be enhanced by the presence of free nucleotides such as inosine-5′-monophosphate (IMP), guanosine-5′-monophosphate (GMP) and adenosine-5′-monophosphate (AMP) [53]. Glutamate is one of the most abundant naturally occurring amino acids and free glutamic acid is found in many foods, such as meat and poultry, seafood, seaweed, cheese, fermented beans, tomato and mushrooms [54]. Several Asian foods—for example, soy sauce and fish sauce—are rich in umami compounds [55]. The cooking temperature and time may affect the concentration of umami compounds in pork meat [52]. 

Natural umami sources have been used in salt-reduced foods—for example, mushrooms in meat dishes [56]. Soy sauces contain salt, but it is also possible to reduce the salt content of a product by replacing NaCl with soy sauce, without lowering the total taste intensity and pleasantness [57]. Monosodium glutamate (MSG) is often used as a flavor enhancer in savory foods. The addition of MSG to spicy soups allowed the reduction of the sodium content without affecting the pleasantness, saltiness or taste intensity of the soups [58]. Calcium di-glutamate (CDG) may also improve the sensory and hedonic characteristics of lower sodium foods because it was found that CDG could partly replace sodium chloride in chicken broths [59].

### 3.5. Modification of Food Matrix and Texture

Food matrix and texture may have effects on sodium release and, thus, saltiness perception and may offer new options for salt reduction [60]. Product composition examples are the rate of dissolution from salt crystals via changing their size and shape, textural effects such as the hardness/brittleness of the food product and inhomogeneous distribution of salt to provide taste contrasts [61]. For example, using coarse-grained NaCl in bread significantly accelerated sodium release and led to enhanced salt taste and a sodium reduction in bread by 25% while maintaining taste [62]. Additionally, the late addition of coarse-grained NaCl to pizza dough was shown to enhance saltiness through taste contrast and accelerated sodium delivery in the mouth [63]. 

Modification of wheat bread texture resulted in a significantly faster sodium release from coarse-pored compared with fine-pored bread, thus enhancing saltiness perception [64]. Air inclusions within hydrogels were shown to increase both the delivery and perception of salt and aroma [65]. Interestingly, sandwiches containing regions of different salt levels were perceived significantly saltier than sandwiches containing the same overall salt content distributed homogeneously, suggesting that perceptual expectation based on the first bite can influence saltiness perception [66]. Heterogeneous salt distribution in hot-served layered snack foods was also observed to enhance saltiness perception [67].

### 3.6. Odor-Taste Interactions

Additionally, cross-modal odor-taste interactions could be applied in the context of sodium reduction in complex food systems. Nasri et al. [68] reported that the addition of sardine aroma to the salt-containing solution compensated for a decrease of 25% of the salt content. Higher levels of savory aroma were also shown to compensate for salt reduction in instant bouillons [69]. Lawrence et al. [70] investigated odor-induced saltiness enhancement in a solid model cheese and showed that salt-associated odors (e.g., comté cheese) could enhance saltiness perception. 

On the other hand, in a cheese flavor model (varying aroma, NaCl and lactic acid levels), it was shown that the levels of the tastes could be manipulated only to a certain extent before the cheese flavor intensity was suppressed [71]. Furthermore, Linscott and Lim [72] reported that saltiness and umami enhanced chicken and soy sauce odor intensities, while odors did not enhance taste intensities. Therefore, odor-taste interactions are challenging and require further study.

### 3.7. Herbs and Spices 

Herbs and spices are often recommended to use in salt reduction [73], but there are very few studies focusing on this issue from a sensory point of view. Wang et al. [74] reported that the amount of salt consumers added to soup was decreased when the perceived herb flavor increased. However, high levels of herbs decreased the overall liking of soups [74]. An herb and spice blend added to tomato soup was found to enhance the perception of salty taste of the low-salt soup, and repeated exposure to the soups with herbs and spices increased their overall liking [75]. 

### 3.8. Flavor Peptides

Food protein-derived peptides are, in addition to the nutritional properties, important factors for the taste of processed and unprocessed foods [76,77]. Depending on the structure, peptides can elicit salty, sweet, sour, bitter and umami taste modalities and/or induce flavor-enhancing effects, while some peptide structures are neutral in taste [76,77,78]. Flavor peptides are typically less than 3000 Da in molecular weight, and the interaction in taste is enabled with polar groups, amino and carboxyl groups [76]. Various food protein-derived hydrolysates and peptides have shown salty and umami flavors—for example, proteins from beef, fish, chicken, soy and nuts have been used to produce peptides with umami and salty flavor [77,78,79,80,81,82]. Salty flavor is attributed to the presence of charged terminals and amino acid residues in the peptide structure. Thus, the zwitterionic nature of the peptide is more important for the salty flavor than for the general conformational features of the peptide. 

Despite the potential of peptides for the modification of flavor, the utilization of flavor peptides for salt reduction in food products is not straightforward. The concentration of unique flavor peptide structures in food matrixes and protein hydrolysates is typically low. Thus, the targeted peptide structures with salty flavor should be concentrated effectively before application into food products. Feasible technological solutions are needed to enable the concentration. Additionally, the safety of the concentrated peptide fractions needs to be considered and evaluated before application into food products.

### 3.9. Food Services

The above salt reduction of single food items in the food market has been covered, but much less is known about the content of salt and efforts to reduce it in various food services. Workplace canteens, restaurants and fast-food outlets are offering various options for their customers, but the sodium content of many food items and meals seems too high [83,84]. Furthermore, the trends do not seem to be improving because, between 1997/1998 and 2009/2010 at leading fast-food restaurants in the USA, the sodium content of lunch/dinner menu items has increased by 23% [85]. Customers have been found to substantially underestimate the sodium content of meals in fast-food restaurants [86].

Few studies have focused on consumer acceptance of reduced-sodium foods in catering services. In an experimental real-life canteen study setting, the consumption of reduced-sodium lunches was well accepted by consumers and decreased daily sodium intake [87]. In the USA, reducing sodium in restaurant menu items showed that the majority of slight to moderate versions of sodium-reduced items were acceptable to consumers [88]. A questionnaire study on consumer attitudes and meal satisfaction concerning sodium-reduced meals at worksite cafeterias suggested that improving the taste and diversifying the menus could lead the consumers to choose sodium-reduced meals [89]. 

Customers reported to be willing to see information on the salt content of the menu items in catering services [90] and that could assist consumers to make healthy choices. Restaurant owners and chefs seem to have mixed opinions. A survey among US take-out restaurant owners and chefs has indicated that most of them were willing and able to reduce the sodium content of their meals, but they also wanted to have training in food preparation and marketing of low-salt dishes [91]. On the other hand, food service chefs/managers in the UK and USA were found to be reluctant to reduce salt use and feared negative business outcomes along with salt reduction [92].

### 3.10. Challenges in the Sensory Evaluation of Reduced-Salt Products

As the previous examples have demonstrated, the sensory evaluation and consumer liking of salt-reduced products are a great challenge. As the results have been shown to be related to other sensory characteristics of the product, different salt reduction options may work in different products—for example, bread and meat products [39]. Because the salt content may also affect, for example, texture characteristics and color [35], the evaluation of salty taste intensity is usually not sufficient, but other sensory characteristics must be included.

Considering the practical aspects of sensory evaluation, the use of a trained panel or consumer tests should be carefully planned. Trained panels can evaluate small differences in sensory quality and are needed in the initial phase of screening salt reduction options. However, consumer evaluations and preferences are important to obtain an overview of the market potential. A useful approach is to apply both a trained panel and consumers together. Different consumer groups should be represented in the tests—for example, gender, age, and smoking may be associated with salty food preferences [93]. The challenge currently is that consumers have so many different lifestyle variables, values, attitudes, and motives, characteristics that might affect their food and product preferences [94,95]. 

The results obtained in the sensory laboratory may also differ from those obtained under real tasting conditions, such as home experiments. Romagny et al. [96] employed different methodologies and combined home and laboratory evaluations of pleasantness and willingness to pay to evaluate the impact of reducing fat, salt and sugar in commercial food products. They found that, in most cases, the reformulated products maintained consumer acceptance. Willems et al. [97] studied repeated in-home consumption on the liking of reduced-salt soups (regular-salt soup compared with 22% and 32% salt-reduced soups) and found no difference in liking the soups when consumed at home (twice weekly for five weeks). Furthermore, the initial liking was not predictive of liking after repeating the in-home period.

Herbert et al. [98] introduced a new method to quantify memory for the sensory characteristics of a recently consumed food. They found that most people recalled a reduced-salt soup as having a higher salt concentration and suggested that remembered saltiness is influenced by representations of ideal saltiness. They concluded that salt concentrations could be reduced to a greater extent than might be predicted by a direct comparison between a regular and a reduced-salt product [98].

## 4. Conclusions

Along with the public health targets and responsibility demands, it is clear that the food industry, restaurants and catering services should provide more low-salt options for consumers. Reducing salt is a challenge for food development because, besides flavor, issues such as texture and microbial safety must be targeted. The food industry and researchers often have a technological starting point to new innovations, but many consumers may have critical views toward, for example, new food additives [99]. Currently, many consumers prefer natural ingredients [100]. Thus, consumer perceptions on the acceptability of new salt taste replacers/modifiers should be evaluated well in advance in the development process.

With the vast supply of products in the market, it may be difficult to find low-salt products and, for many consumers, understanding the package markings and nutrient content information in food labels may be challenging. Therefore, clear labeling of lower salt products would be necessary. However, health labeling may have a negative effect on taste perception [101]. This finding indicates that consumer preferences of packaging and labeling options are also important to study. 

Additionally, actions targeting consumer attitudes and knowledge on salt reduction are needed. The general population may not be aware of the salt intake recommendations and salt content of foods, and may not be interested in salt reduction [102]. Not even hypertension patients are aware or reach sodium targets [103]. Additionally, other healthy dietary habits are important for cardiovascular health [104]. 

In conclusion, many new options for products with a lower sodium content are available. However, evaluating consumer preferences of reduced salt products are of crucial importance for their market success. Raising consumer awareness of the need to reduce salt intake through social marketing and promotion of healthy food options in schools, workplaces, and other communities is important. A close collaboration between experts in food chemistry, food technology, sensory science, nutrition, and consumer science, as well as practical experts in the food industry and food services, is needed to cover consumer expectations of high-quality, healthy and good-tasting products.
